# Hospital-based interventional two-arm parallel comparative study on dydrogesterone vs combined oral contraceptive pills for functional ovarian cysts

**DOI:** 10.12688/f1000research.142012.1

**Published:** 2024-01-25

**Authors:** Muneeba Shaikh, Neema Acharya

**Affiliations:** 1 Obstetrics and Gynaecology, Datta Meghe Institute of Higher Education & Research, Wardha, Maharashtra, 442001, India

**Keywords:** Functional ovarian cysts, Combined oral contraceptive pills, Dydrogesterone, Reproductive-age women, Efficacy, Safety

## Abstract

**Background:**

Functional ovarian cysts are common among women of reproductive age, often necessitating medical intervention. This hospital-based interventional study compares the efficacy and safety of combined oral contraceptive pills (COC) and dydrogesterone in managing functional ovarian cysts.

**Methods:**

This randomized controlled trial will be conducted over two years at the Department of Obstetrics & Gynecology, AVBRH, Datta Meghe Institute of Medical Sciences. The study population consists of reproductive-age women seeking care at the outpatient unit of Obstetrics and Gynecology at AVBRH hospital. The sample size of 46 participants per group has been calculated based on a 95% confidence interval and the estimated prevalence of functional ovarian cysts. Group A will receive low-dose COC for three menstrual cycles. At the same time, Group B will be administered dydrogesterone (10 mg twice daily) for ten days during the luteal phase, repeated across three cycles.

**Expected outcomes:**

The primary outcomes include evaluating the recession of cysts within three months, monitoring alterations in menstrual patterns (frequency, regularity, duration, and volume), assessing the necessary treatment duration, and observing potential side effects (e.g., nausea, vomiting, weight gain, and acne) and complications (e.g., thromboembolism, delayed menstrual cycles post-treatment, and interactions with other drugs). Data analysis will encompass descriptive statistics, comparative tests, and regression models to assess the primary outcomes. The significance level for hypothesis testing will be 0.05 with a two-tailed approach.

**Registration:**

CTRI/2023/04/051811.

## Introduction

Functional ovarian cysts, a prevalent occurrence among women of reproductive age, frequently require medical intervention due to their potential to disrupt fertility and overall health. These cysts originate from the intricate interplay of normal physiological processes involved in ovulation and follicular development. ^1^ However, in certain instances, deviations from this intricate balance can lead to the development of cysts that warrant medical attention. Such deviations can manifest as variations in size, persistence, or symptomatic expression, prompting clinical intervention to prevent complications and alleviate patient discomfort.

The management landscape for functional ovarian cysts comprises a spectrum of approaches, encompassing both surgical and medical modalities. Surgical interventions, while effective, often involve inherent risks and potential disruptions to reproductive function. On the other hand, medical options promise to preserve fertility and avoid invasive procedures. Despite the existing armamentarium, pursuing treatments that balance efficacy and safety remains an ongoing endeavor. ^2^^,^^3^

This study seeks to address this need by conducting a hospital-based interventional two-arm parallel comparative investigation to compare the effectiveness and safety of two distinct medical interventions – combined oral contraceptive pills (COC) and dydrogesterone. COC, renowned for their hormonal regulatory effects, have demonstrated potential in mitigating cystic growth. Conversely, dydrogesterone, a synthetic progestin, presents an alternative therapeutic avenue with its luteal phase support capabilities. ^4^

The primary objective is to evaluate the efficacy of these interventions in terms of cyst regression and their influence on the menstrual pattern. Additionally, the study will focus on assessing the safety profile, including potential side effects, treatment duration, and the occurrence of complications. The outcomes of this research will contribute essential insights to inform clinical decision-making, guide treatment strategies, and optimize patient care. By comparing the outcomes of these interventions in a controlled and systematic manner, this study advances our understanding of medical management approaches for functional ovarian cysts, ultimately enhancing the quality of care for women facing this common medical concern.

### Aim

To compare the effectiveness and safety of combined oral contraceptive pills (COC) and dydrogesterone for the medical management of functional ovarian cysts.

### Objectives

1. To study the effectiveness and safety of combined oral contraceptive pills in treating functional ovarian cysts.
2. To study the effectiveness and safety of dydrogesterone in treating functional ovarian cysts.
3. To compare the effectiveness and safety between combined oral contraceptive pills and dydrogesterone.

## Protocol

### Study design

This research employs a randomized controlled trial methodology, falling within the category of interventional studies. The study is planned to span a duration of 2 years. The study will be conducted at the Department of Obstetrics & Gynecology, AVBRH, Datta Meghe Institute of Medical Sciences, in Sawangi (Meghe), Wardha.

### Participants

The study will focus on women of reproductive age seeking medical attention at the outpatient unit of Obstetrics and Gynecology within AVBRH hospital. These participants will be selected based on their fulfillment of the defined inclusion and exclusion criteria.

### Inclusion criteria

Participants within the reproductive age range of 18 to 44 years, possessing ovarian cysts between 3 to 10 centimeters with a single compartment and precise contents, and having a body mass index (BMI) within the range of 18.5 to 29.9 will be considered for inclusion.

### Exclusion criteria

Prospective participants with dermoid cysts, endometriosis, malignancies, ruptured cysts, cyst torsion, recent use of hormonal medications, history of ovarian cyst removal (cystectomy), and specific medical conditions will be excluded from the study.

### Allocation

The eligible participants will be assigned randomly to two study groups utilizing a 1:1 allocation ratio. The random allocation will be achieved by applying the sealed envelope technique.

### Interventions

Participants in Group A will receive a course of ethinyl estradiol (0.03 mg) + Levonorgestrel (0.15 mg) combined oral contraceptive pills over three menstrual cycles. In Group B, participants will be administered dydrogesterone, with 10 mg taken twice daily for ten days during the luteal phase. This protocol will be repeated across three consecutive menstrual cycles.

Criteria for discontinuing or modifying allocated interventions will be clearly defined in our study. These criteria may include, but are not limited to, the development of severe adverse events, non-compliance with the treatment protocols, or the emergence of conditions that contraindicate further participation. In such cases, the participant will be withdrawn from the study, and the reasons for discontinuation or modification will be documented.

To ensure participant adherence to allocated interventions, we will implement the following strategies:

• Regular follow-up appointments and reminders to participants regarding treatment regimens.
• Monitoring of participant compliance through self-reporting and pill counts for both intervention groups.
• Tracking participant satisfaction and addressing any concerns or issues that may affect adherence.
• Continuous communication between the research team and participants to address any questions or issues related to the interventions.

Participants will be advised to avoid the use of other hormonal medications or treatments during the study period, as these may confound the results. However, the use of non-hormonal over-the-counter medications will be allowed as needed, and these instances will be recorded.

### Enrolment

We will employ several strategies to achieve adequate participant enrolment:

• Collaboration with healthcare providers in the Department of Obstetrics & Gynecology, AVBRH, to facilitate referrals and patient recruitment.
• Public awareness campaigns and information dissemination about the study in the hospital’s outpatient unit.
• Ensuring that the inclusion and exclusion criteria are clear and well-communicated to potential participants.

### Allocation

The allocation sequence will be generated by a biostatistician, who is not directly involved in the enrolment process. The enrolment of participants will be conducted by clinical research coordinators, while the assignment of participants to interventions will be performed by a research pharmacist based on the allocation sequence. Due to the nature of the interventions, participants cannot be blinded. However, outcome assessors and data analysts will be blinded to the treatment assignments. Unblinding may be considered in the event of a medical emergency or if knowledge of the treatment assignment is deemed necessary for the safety and well-being of the participant. Unblinding will only occur when the unblinded status is required for clinical management.

### Follow-up

We will implement the following plans to promote participant retention and complete follow-up:

• Regular follow-up appointments and communication to encourage participants to complete the study.
• Collecting contact information and maintaining updated records for all participants.
• Clearly outlining the importance of continued participation and the potential consequences of non-completion at the informed consent stage.

## Methods: monitoring

A Data Monitoring Committee is not deemed necessary for this study due to its relatively low-risk nature and the absence of interim analyses or stopping guidelines. Interim analyses are not planned for this study, and as such, there are no specific stopping guidelines. The final decision to terminate the trial will be made by the principal investigator in consultation with the research team.

### Adverse events and unintended effects

We will implement systematic procedures for collecting, assessing, reporting, and managing adverse events. Adverse events will be documented by the clinical research team, assessed for causality and severity, and reported to the appropriate regulatory authorities as per regulatory requirements.

### Auditing trial conduct

An independent auditing process for trial conduct will not be conducted, as the study is not expected to pose significant risks to participants. However, regular internal quality control checks and monitoring of data integrity will be performed throughout the study to ensure data accuracy and adherence to the protocol.

### Primary outcome measures

**Effectiveness assessment:**

1. **Regression of cysts:** To monitor the regression of ovarian cysts, we will conduct regular ultrasound evaluations. Participants from both study groups will undergo ultrasound assessments after each menstrual cycle. These assessments will continue until either the cysts dissipate or for a maximum duration of three months.
2. **Menstrual pattern changes:** To observe shifts in menstrual patterns, we will have participants maintain menstrual diaries. Participants will record information regarding the frequency, regularity, duration, and volume of their menstrual cycles. Data will be collected at each follow-up appointment, allowing us to track any changes over time.

**Safety assessment:**

1. **Duration of treatment:** We will determine the duration of treatment necessary for each participant by monitoring their response and progress during follow-up appointments. The treatment duration will be based on individual responses and the predefined treatment protocol.
2. **Monitoring side effects:** Participants will be encouraged to report any potential side effects during regular follow-up appointments and will also have access to a dedicated contact point for immediate reporting. Adverse events, including nausea, vomiting, weight gain, and acne, will be documented and assessed for severity and causality.
3. **Effects on liver function tests:** We will conduct regular blood tests to assess liver function. Liver function tests, including alanine aminotransferase (ALT) and aspartate aminotransferase (AST) levels, will be measured at specific intervals during the study. Any abnormal results will be monitored closely.
4. **Tracking critical complications:** We will monitor for critical complications such as thromboembolism, delayed menstrual cycles post-treatment, and potential interactions with other drugs through continuous review of participants’ medical records and regular assessments during follow-up appointments. In the event of a critical complication, immediate actions will be taken following our predefined clinical management plan.

### Follow-up

An ultrasound evaluation will be integrated into the study’s follow-up process. Participants enrolled in both study groups will undergo thorough ultrasound assessments after each menstrual cycle. This evaluation will continue until either the cysts dissipate or for a maximum duration of three months.

### Sample size

Formula using mean difference

$$n1=n2=2\frac{{\left(Z_{\alpha }+Z_{\beta }\right)}^{2}\sigma^{2}}{{\left(\delta \right)}^{2}}$$

$$Z_{\alpha }=1.96$$

α=Type I error at 5% at both sides two tailed

$$Z_{\beta }=1.28=\text{Power at 90\%}$$

Dydrogesterone vs combined oral contraceptive pills for functional ovarian cysts (δ) = 0.3

Considering 30% superiority = (0.3*30)/100 = 0.09

Standard deviation = 1

Minimum sample size N = $n1=n2=2\frac{{\left(1.96+0.09\right)}^{2}{\left(\;1\right)}^{2}}{{\left(0.09\right)}^{2}}=42\;\text{per group}$

Considering 10% drop out = 4

Total sample size required = 42 + 4 = 46 per group. Reference study. ^5^

### Statistical analysis

The statistical analysis plan for this study will involve several key steps. Descriptive statistics will be used to summarize baseline characteristics of participants in both treatment groups. Continuous variables will be presented as means with standard deviations or medians with interquartile ranges, while categorical variables will be summarized as frequencies and percentages.

For the primary outcomes, the effectiveness and safety of the two treatments will be assessed separately. The regression of cysts within the three-month timeframe will be compared between groups using appropriate statistical tests, such as the Chi-square test or Fisher’s exact test. Changes in the menstrual pattern will be analyzed using paired t-tests or non-parametric tests if assumptions are not met.

Safety outcomes, including the duration of treatment needed and the incidence of side effects, will be compared using appropriate statistical tests, depending on the distribution of data. The impact on liver function tests and the occurrence of serious complications will be analyzed using appropriate statistical methods, potentially logistic regression, or survival analysis for time-to-event outcomes.

A comparative analysis between the two treatment groups will be performed using appropriate statistical tests, such as t-tests, Mann-Whitney U tests, or chi-square tests, depending on the nature of the data. Adjustments for potential confounders, such as age, BMI, and baseline cyst characteristics, will be considered using regression models. The significance level for hypothesis testing will be set at 0.05, and all tests will be two-tailed. Confidence intervals will be calculated where relevant.

## Discussion

The present study protocol outlines a comprehensive framework for investigating the efficacy and safety of combined oral contraceptive pills (COC) and dydrogesterone in managing functional ovarian cysts. The study’s design and methodology reflect a systematic approach to addressing the clinical challenges posed by these cysts while contributing to evidence-based treatment decisions.

**Functional ovarian cysts and clinical significance:** Functional ovarian cysts are common among reproductive-aged women, often necessitating medical attention due to their potential to disrupt fertility and overall well-being. These cysts arise from the complex interplay of hormonal fluctuations and follicular development, with deviations from the norm occasionally resulting in cysts that require clinical intervention. The clinical significance lies in the potential for these cysts to cause discomfort, pain, and menstrual irregularities, thereby impacting the quality of life and reproductive potential of affected individuals. ^6^

**The rationale for study design:** The chosen study design, an interventional two-arm parallel comparative study, is apt for evaluating the relative efficacy and safety of COC and dydrogesterone. This design allows for direct comparison between the two treatments, enabling researchers to draw meaningful conclusions about their merits. By employing random allocation and well-defined inclusion and exclusion criteria, the study minimizes bias and enhances the validity of the results. ^7^

**Treatment options and therapeutic landscape:** The study’s focus on COC and dydrogesterone is well-founded, as both interventions have demonstrated potential in addressing functional ovarian cysts. With their hormonal regulation properties, COC may modulate the hormonal milieu to suppress cystic growth. Dydrogesterone’s specific luteal phase support properties offer an alternative mechanism of action, potentially influencing cyst resolution. This dual intervention approach enriches the understanding of treatment possibilities. ^8^^,^^9^

**Primary outcome measures:** The primary outcomes, centered around effectiveness and safety, encompass the regression of cysts, alterations in menstrual patterns, treatment duration, side effects, and complications. These outcomes holistically capture the impact of the interventions on both the physical and overall well-being of participants. Monitoring cyst regression and menstrual pattern changes offers insights into the interventions’ therapeutic effects, while evaluating safety profiles contributes to a comprehensive understanding of potential risks.

**Implications and future directions:** The findings of this study have far-reaching implications for clinical practice. The data generated from the study will aid clinicians in making informed treatment decisions, balancing effectiveness and safety considerations. Furthermore, this research sets the stage for more targeted investigations into the mechanisms of action of COC and dydrogesterone, potentially uncovering novel insights into the pathophysiology of functional ovarian cysts.

**Limitations and considerations:** It’s important to acknowledge potential limitations, such as the limited follow-up duration and the exclusive focus on two treatment options. Future studies could explore the effects of alternative interventions and longer-term outcomes, providing a more comprehensive understanding of functional ovarian cyst management.

### Ethical considerations

The Institutional Ethics Committee of Datta Meghe Institute of Higher Education and Research (DU) has granted its approval to the study protocol, dated 21/07/22, reference DMIHER (DU)/IEC/2022/115. Prior to commencing the study, we will obtain written informed consent from all participants, providing them with a comprehensive explanation of the study’s objectives. We will prioritize the interviewee’s privacy and comfort during the interview process. This protocol has also been registered with CTRI: CTRI/2023/04/051811.

### Dissemination

This study protocol will be published in an indexed journal.

### Study status

The study has not yet started, we will start after publishing the study protocol.

## Data Availability

No data are associated with this article.
