# Supplementary material for: Hospital-based interventional two-arm parallel comparative study on dydrogesterone vs combined oral contraceptive pills for functional ovarian cysts
Source: F1000Res. 2024 Jan 25;13:84. [Version 1] doi: 10.12688/f1000research.142012.1 (PMC10912786; doi:10.12688/f1000research.142012.1)
Supplement: Peer review history

# Reviewer report: Reviewer response for version 1

**DOI:** 10.5256/f1000research.155504.r245736

**Published:** 2024-03-04

**Authors:** Sandhya Gadre

**Affiliations:** 1 Department of Obstetrics and Gynecology, Chirayu Medical College and Hospital, Bhopal, India

The study deals with functional ovarian cysts, which is seen in reproductive age group. The objective of the study is to compare the efficacy and safety of combined oral contraceptive pills (COC) and dydrogesterone in managing functional ovarian cysts.

 It is proposed that a randomized controlled trial will be conducted over a period of  two years.  In each group 46 subjects would be included. One group will receive low-dose COC ethinyl estradiol (0.03 mg) + Levonorgestrel (0.15 mg)  for three menstrual cycles. Other Group will be given dydrogesterone  10 mg twice a day  for ten days during the luteal phase, which will be repeated for three cycles. Random allocation of the study participants will be done using 1:1 allocation ratio, using sealed envelopes.

 The researchers expect the cysts to regress in three months, menstrual pattern would also get altered. The subjects will be monitored for potential side effects & complications of the interventional drugs.

  The data analysis is planned  with the help of  descriptive statistics, comparative tests, and regression models for assessment of primary outcomes. The significance level for hypothesis testing will be 0.05.

 Adverse events and unintended effects will be documented by the clinical research team, assessed for causality and severity, and reported to the appropriate regulatory authorities.

 The effectiveness will be assessed by the regression of cysts and menstrual pattern change. The safety would be assessed with the help of duration of treatment, monitoring the side effects,  effects on liver function tests & tracking critical complications. Monitoring of the cysts is planned by ultrasound on a monthly basis. 

 The statistical analysis involves many key steps. Baseline characteristics of participants in both treatment groups will be summarized using Descriptive statistics. Continuous variables will be presented as means with standard deviations or medians with interquartile ranges and categorical variables will be summarized as frequencies and percentages. The regression of cysts will be compared between groups using Chi-square test or Fisher’s exact test. Changes in the menstrual pattern will be analyzed using paired t-tests or non-parametric tests. Safety outcomes,  impact on liver function tests and the occurrence of serious complications will be compared using appropriate statistical tests. 

 A comparative analysis between the two treatment groups will be performed using, such as t-tests, Mann-Whitney U tests, or chi-square tests, depending on the nature of the data. Adjustments for potential confounders, will be considered using regression models. 

 All the ethical considerations will be taken care of.

**Strengths of the Study :**

**Major Points**

 1.The effectiveness & safety profile, both effects are considered for the intervention planned, with various assessment parameters reflecting the positivity of the study.

 2.Sufficient details of the methods are provided in a way it can be replicated by other researchers. 

 3.For different assessment parameters, different statistical analysis methods are thoughtfully planned.

 4.The proposal is worth approving for study. 

**Minor Points**

****

 1.The objectives of the study are clearly described

 2.The study design is appropriate for the research question.

 3.The study deals with a problem which is faced routinely in clinical practice. The conclusion of the completed study may prove beneficial for practical purposes.

 I do not advice any changes in the study.

Is the study design appropriate for the research question?

Yes

Is the rationale for, and objectives of, the study clearly described?

Yes

Are sufficient details of the methods provided to allow replication by others?

Yes

Are the datasets clearly presented in a useable and accessible format?

Not applicable

Reviewer Expertise:

Subjects of clinical orientation.

I confirm that I have read this submission and believe that I have an appropriate level of expertise to confirm that it is of an acceptable scientific standard.

## References

[1] : Hospital-based interventional two-arm parallel comparative study on dydrogesterone vs combined oral contraceptive pills for functional ovarian cysts. *F1000Research* .2024;13: 10.12688/f1000research.142012.1 10.12688/f1000research.142012.1 PMC1091278638450050

[2] : Efficacy and safety of chimeric antigen receptor T-cell (CAR-T) therapy in hematologic malignancies: a living systematic review on comparative studies. *Ther Adv Hematol* .2023;14: 10.1177/20406207231168211 20406207231168211 10.1177/20406207231168211 37138698 PMC10150428

# Reviewer report: Reviewer response for version 1

**DOI:** 10.5256/f1000research.155504.r245739

**Published:** 2024-02-13

**Authors:** Nilaj Bagde

**Affiliations:** 1 Department of Obstetrics and Gynecology, All India Institute of Medical Sciences, Raipur, Chhattisgarh, India; 2 Gynecology, AIIMS Raipur, Raipur, Chattisgarh, India

The study design is appropriate. The justification and background are adequately presented. The study objectives are clear and in measurable terms. Randomization method, The method of generation of allocation is clear and appropriate. Sample size with statistical formula is appropriate for study design statistical Methodology, Data collection and statistical analysis have been written sufficiently and clearly.

Is the study design appropriate for the research question?

Yes

Is the rationale for, and objectives of, the study clearly described?

Yes

Are sufficient details of the methods provided to allow replication by others?

Yes

Are the datasets clearly presented in a useable and accessible format?

Not applicable

Reviewer Expertise:

obstetrics and Gynecoloy, Infertility, endometriosis, Gynecology endoscopy, cancer cervix

I confirm that I have read this submission and believe that I have an appropriate level of expertise to confirm that it is of an acceptable scientific standard.
